# Trace Level Determination of Mesityl Oxide and Diacetone Alcohol in Atazanavir Sulfate Drug Substance by a Gas Chromatography Method

**DOI:** 10.3797/scipharm.1507-05

**Published:** 2015-08-27

**Authors:** K. V. S. N. Raju, K. S. R. Pavan Kumar, N. Siva Krishna, P. Madhava Reddy, N. Sreenivas, Hemant Kumar Sharma, G. Himabindu, N. Annapurna

**Affiliations:** 1 Aurobindo Pharma Limited Research Centre-II, Survey No. 71&72, Indrakaran Village, Sangareddy Mandal, Medak-502329, Andhra Pradesh, India; 2 Department of Engineering Chemistry, AU college of Engineering, Andhra University, Visakhapatnam-530003, Andhra Pradesh, India

**Keywords:** Mesityl oxide, Diacetone alcohol, Atazanavir sulfate, GC-FID, Development, Validation

## Abstract

A capillary gas chromatography method with a short run time, using a flame ionization detector, has been developed for the quantitative determination of trace level analysis of mesityl oxide and diacetone alcohol in the atazanavir sulfate drug substance. The chromatographic method was achieved on a fused silica capillary column coated with 5% diphenyl and 95% dimethyl polysiloxane stationary phase (Rtx-5, 30 m x 0.53 mm x 5.0 µm). The run time was 20 min employing programmed temperature with a split mode (1:5) and was validated for specificity, sensitivity, precision, linearity, and accuracy. The detection and quantitation limits obtained for mesityl oxide and diacetone alcohol were 5 µg/g and 10 µg/g, respectively, for both of the analytes. The method was found to be linear in the range between 10 µg/g and 150 µg/g with a correlation coefficient greater than 0.999, and the average recoveries obtained in atazanavir sulfate were between 102.0% and 103.7%, respectively, for mesityl oxide and diacetone alcohol. The developed method was found to be robust and rugged. The detailed experimental results are discussed in this research paper.

## Introduction

Atazanavir sulfate is chemically known as (3*S*,8*S*,9*S*,12*S*)-3,12-bis(1,1-dimethylethyl)-8-hydroxy-4,11-dioxo-9-(phenylmethyl)-6-[[4-(2-pyridinyl)phenyl]methyl]-2,5,6,10,13-pentaazatetradecanedioic acid 1,14-dimethyl ester sulfate (1:1). Its molecular formula is C_38_H_52_N_6_O_7_ H_2_SO_4_ and its molecular weight is 802.93. It is an azapeptide and is the 7^th^ protease inhibitor [1] used in the treatment of human immunodeficiency virus (HIV) type II infection. Atazanavir is a commonly used HIV protease inhibitor and is used in combination with other antiretroviral agents for the treatment of HIV infection. The efficacy of atazanavir has been assessed in a number of well-designed trials in ART-naive and ART-experienced adults [2]. Atazanavir has been approved in the European Union for once-daily administration of 300 mg in combination with 100 mg of ritonavir. The protease inhibitor ritonavir is given as a pharmacokinetic booster and increases systemic atazanavir exposure by inhibiting cytochrome P450 enzyme 3A4 (CYP3A4) metabolism in the liver and intestines. In the United States, atazanavir has also been approved in a dose of 400 mg once daily without ritonavir in treatment-naive patients [3]. It is marketed under the trade name Reyataz by the Bristol-Myers Squibb pharmaceutical company [4]. Residual solvents in pharmaceuticals are used in the manufacture of drug substances which are to be evaluated and should be removed or controlled to the greatest extent possible as they do not provide therapeutic benefits and they are not completely removed by practical manufacturing techniques [5].

**Fig. 1:**
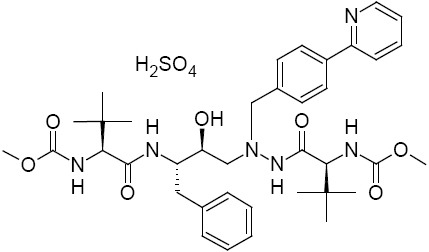
Chemical structure of atazanavir sulfate

**Fig. 2:**
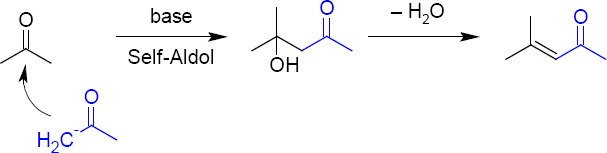
Mechanism for the formation of diacetone alcohol and mesityl oxide from acetone.

Mesityl oxide (MO) is an α,β-unsaturated ketone with the formula CH_3_C(O)CH=C(CH_3_)_2_ and diacetone alcohol (DO) is a chemical compound with the formula CH_3_C(O)CH_2_C(OH)(CH_3_)_2_. These two residual impurities are present in commercial grade acetone at trace levels. A common use of acetone is as a solvent, which is a substance that is capable of dissolving another substance. It is a popular solvent for many plastics and synthetic fibers and the same is used in the final crystallization step of ATV. In basic medium, DO will be formed from acetone, and if a water molecule is lost from DO, MO will appear and these two impurities may carry to the final drug product. MO contains an α, β-unsaturated ketone moiety which presents a structural alert for genotoxicity, however. Since mesityl oxide possesses a conventional α, β-unsaturated ketone structural alert, and is often identified as a potential genotoxic impurity in drug substances that have been crystallized from acetone, it is a potential impurity in this solvent. Although, mesityl oxide is reported to be Ames-negative [6]. The mechanism for the formation of DO and MO from acetone is shown in Figure 2 [7].

There are some analytical methods available in literature for the determination of MO and DO. In oilseed meals and flours, these two contents are monitored by GC [8] by using a polarographic method, published in a research paper in 1973 by B. Fleet *et al*. [9]. Till now, no method has been published for MO and DO in atazanavir sulfate. This research paper describes a simple and sensitive gas chromatography method with a flame ionization detector (FID) for the quantification of MO and DO in the ATV drug substance with limit of detection and limit of quantitation values 0.04 and 0.70 µg/ml, respectively. One hundred µg/g has been chosen as the specification level for this research work. The developed method was validated for specificity, sensitivity (limit of detection and limit of quantitation), linearity, precision (system precision, method precision, and intermediate precision), accuracy, and robustness in accordance with ICH guidelines [10].

## Experimental

### Chemicals, Reagents, and Samples

The investigated sample of ATV was gifted from APL Research Centre Laboratories (a division of Aurobindo Pharma Ltd., Hyderabad.). Analytical reagent (AR grade) mesityl oxide, diacetone alcohol, o-xylene, methylene chloride, decane, ethanol, acetone, isopropyl alcohol (2-propanol), methyl tert-butyl ether, benzene, n-heptane, methyl isobutyl ketone (4-methyl-2-pentanone), toluene, and 1-methyl-2-pyrrolidinone were procured from Sigma-Aldrich (Steinheim, Germany). ACS grade formic acid was procured from Merck & HPLC water was procured from Merck, Mumbai, India.

### Equipment and Gas-Chromatographic Conditions

The Agilent 6890N equipped with Flame Ionization Detector with a Gerstel Multipurpose Sampler was used in this research work. Data acquisition and processing were conducted using the HPCHEM station software.

### Operating Conditions for GC

The GC separation was carried out on a Rtx-5 column (make: Restek) with a dimension of 30 m length, 0.53 mm I.D., film thickness 5.0 µm, and the injection volume was 2 µL. The oven temperature gradient started at 80°C and was held for 0 min. Then it was raised to 200°C at the rate of 10°C/min and held at 200°C for 8 min. Helium was used as a carrier gas with a constant pressure of 30 KPa with split mode 1:5. The injector temperature and the detector temperature were kept at 180°C and at 260°C, respectively.

### Preparation of Solutions

#### 75% Formic Acid Solution

Transfer 75 ml of formic acid into a 100-ml clean, dry volumetric flask containing about 10 ml of water, mix it, and then dilute to volume with water.

#### Internal Standard Solution

Accurately weigh and transfer 36 mg of o-xylene and 20 mg of decane into a 50-ml volumetric flask containing about 10 ml of methylene chloride, then dilute to volume with methylene chloride. Further dilute 1.25 ml of this solution to 250 ml with methylene chloride (3.6 µg/ml and 2.0 µg/ml).

#### Preparation of Blank Solution

Transfer 3 ml of internal standard solution into a 10 ml centrifuge tube and add 3 ml of 75% formic acid solution and shake vigorously for 1 min. Allow the two phases to separate. Inject the lower layer (methylene chloride layer).

#### Preparation of Standard Solution (20 µg/ml)

Accurately weigh and transfer 51 mg each of mesityl oxide and diacetone alcohol into a 50-ml volumetric flask containing about 20 ml of internal standard solution, then dilute to volume with the internal standard solution. Further dilute 1.0 ml of this solution to 50 ml with internal standard solution.

Transfer 1 ml of standard solution into a 10-ml centrifuge tube add 2 ml of internal standard solution and add 3 ml of 75% formic acid solution, then shake vigorously for 1 min. Allow the two phases to separate. Inject the lower layer (methylene chloride layer).

#### Preparation of Sample Solution

Accurately weigh and transfer 200 mg of the sample into a 10-ml centrifuge tube, then add 3 ml of 75% formic acid solution. Shake the contents for approximately 5 min. Transfer 3.0 ml of internal standard solution into the centrifuge tube. Again, shake vigorously for 1 min. until the phases separate and the lower layer (methylene chloride layer) can be collected through a disposable pipette and transferred to a 2-ml vial for GC analysis.

## Results and Discussions

### Method Development and Optimization

The challenge is to achieve the detection and quantitation at a low level using the Gas Chromatograph with Flame Ionization Detector (GC-FID) for obtaining good separation and the desired sensitivity. Development trials were initiated with the headspace technique using the stationary phase 6% cynopropyl 94% dimethyl polysiloxane (DB-624; Make: J&W). Each 100 µg/g MO/DO solution was prepared in N,N-dimethylformamide (with the respective sample concentration 100 mg in 1 ml) and the same solution was transferred into the headspace vial and sealed with the help of a screw cap. The vial was incubated at 80°C and injected through an AOC 5000 autoinjector into the GC. In this trial, MO was eluted close to N,N-dimethylformamide (resolution more than 1.0) and DO was eluted after N,N-dimethylformamide with a very low response which may be due to its low volatile nature.

Further different trials were performed by changing the injection technique (headspace to direct injection). In the direct injection technique, the same standard solution and standard solution spiked with sample were injected (2.0 µl) through the AOC 5000 autosampler. In this trial, lot of interference was observed at the retention time of DO, which suggests that another type of sample preparation is required to reduce the interference from the sample matrix for the quantification of MO and DO at the desired sensitivity level.

The extraction technique (a completely dissolved sample in 75% formic acid and analytes extracted with organic solvent) with internal standards have been chosen for low level quantification of MO and DO by a liquid injection technique. *o*-Xylene and decane were chosen as internal standards as these solvents are not utilized in the synthesis of ATV. Methylene chloride was chosen as an extraction solvent. Finally, the Rtx-5 column (30 m x 0.53 mm x 5.0 µm) was used and the Restek column was preferred. As a result, the eluted analyte peaks have good symmetric shapes and retention times. The recovery of MO and DO from the range of ATV investigated, prepared at 10-100 µg/g, was 101–105% for a total of 15 determinations. This recovery was considered satisfactory to meet the stated data quality objective in this investigation. The resolution between MO and DO peaks should be no less than 3.0, and this was kept as system suitability criteria.

### Method Validation

The developed and optimized method was validated for specificity, sensitivity [limit of detection (LOD) & limit of quantitation (LOQ)], linearity, precision [system precision, method precision, and intermediate precision], accuracy, and robustness as per ICH guideline Q2(R1) [10].

#### Specificity

**Fig. 3:**
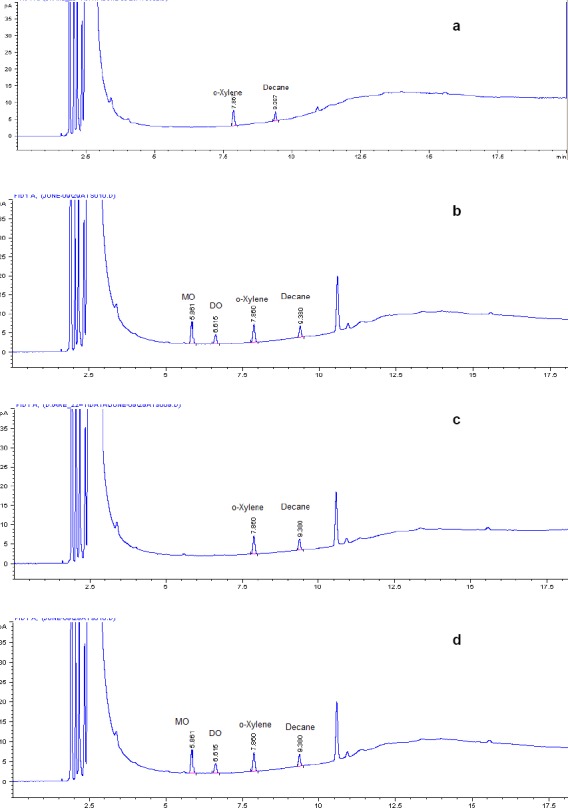
Typical GC chromatograms of a) blank solution, b) standard solution, c) atazanavir sulfate drug substance, and d) atazanavir sulfate drug substance spiked with mesityl oxide and diacetone alcohol.

The blank solution, individual injections of all residual solvents (which are used in the process of ATV), control sample (ATV spiked with MO and DO), and the spiked sample (ATV spiked with MO and DO including other residual solvents) were prepared and injected into the GC and it was found that the MO and DO peaks were well-separated from all other solvents, which indicated that the test method was selective and specific for the determination of MO and DO in ATV. All solvents’ individual retention times are given in Table 1 and the spiked sample data is reported in Table 2. Typical GC chromatograms of the blank solution, standard solution, sample, and spiked sample solutions are shown in Figure 3.

**Tab. 1:**
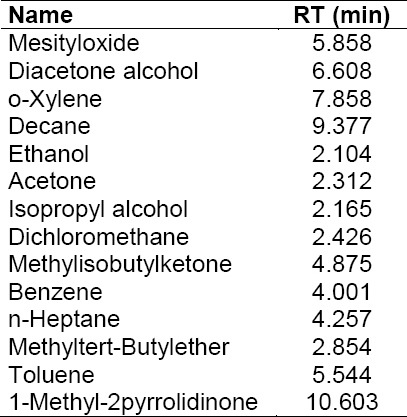
Individual injections of all residual solvents

**Tab. 2:**
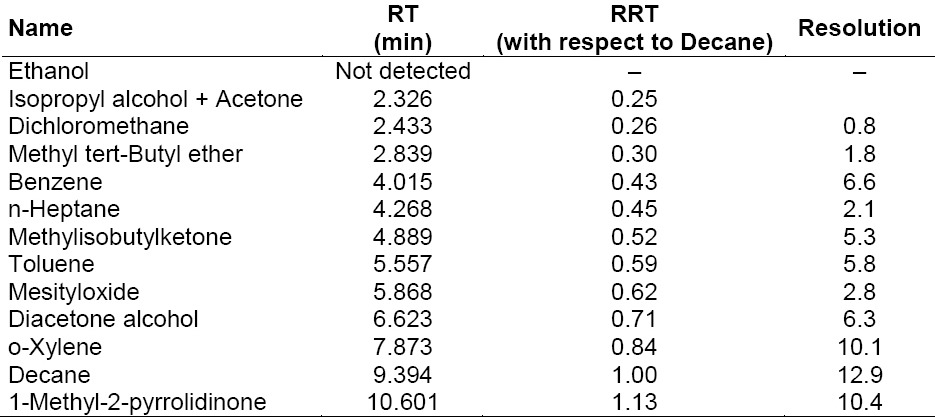
Spiked samples (ATV spiked with MO and DO including other residual solvents)

#### LOD and LOQ

The limit of detection (LOD) and limit of quantification (LOQ) values for MO and DO were determined by the signal-to-noise ratio (s/n) method. The minimum concentration as LOD and the concentration at 10:1 s/n were considered as the LOQ. The LOD and LOQ values obtained for MO and DO were 5 and 10 µg/g, respectively, with respect to sample concentration for both the analytes, which corresponds to 1.04 and 2.08 µg/ml. Precision was verified by preparing the solutions at about the LOD and LOQ concentrations, and injecting each solution six times into the GC and results are tabulated in Table 3.

**Tab. 3:**
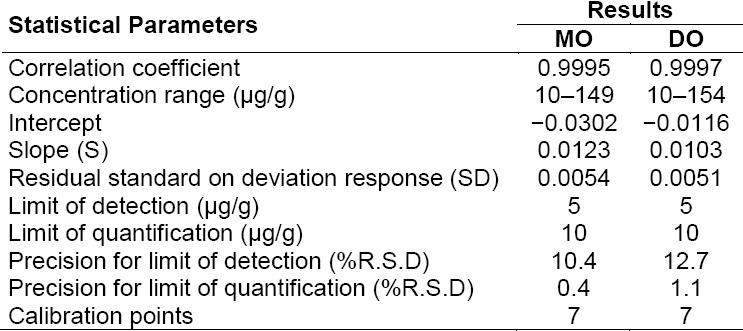
Statistical data of linearity and LOD/LOQ for MO and DO

#### Linearity

The linearity was evaluated by measuring the area ratio for MO and DO with respective internal standards over the range of 2.0 to 30.7 µg/ml [10 to 150 µg/g with respect to sample concentration] and the obtained data was subjected to statistical analysis using a linear regression model. The statistical results like correlation coefficient, slope, intercept, and STEYX are reported in Table 3.

#### Accuracy

The accuracy of the method was verified through performing recovery experiments by spiking known amounts of MO and DO at the LOQ level, 50%, 100%, and 150% of the specification level (i.e. 100 µg/g). The obtained recovery results are tabulated in Table 4, respectively.

**Tab. 4:**
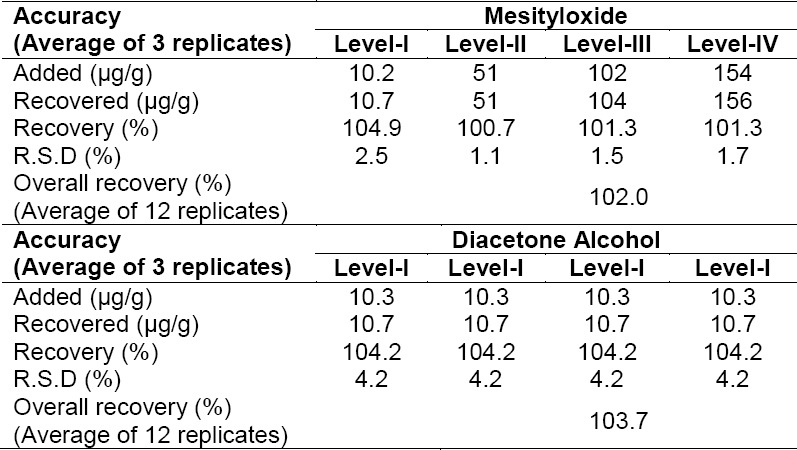
Accuracy data of MO and DO

#### Precision

The system precision was demonstrated by injecting the standard solution of MO and DO six times into the GC and calculating the area ratios using areas obtained from mesityloxide/o-xylene and diacetone alcohol/decane. The method precision was established by preparing six individual sample preparations by spiking MO and DO with the ATV drug substance and injecting into the GC, then calculating the MO and DO contents. The ruggedness of the method was evaluated by preparing six individual sample preparations [the same sample which was used in the method precision experiment] by spiking MO and DO with the ATV drug substance and injecting into the GC and calculating the MO and DO contents using two different columns, a different instrument, and a different analyst on different days. The achieved precision experiment results are reported in Table 5.

**Tab. 5:**
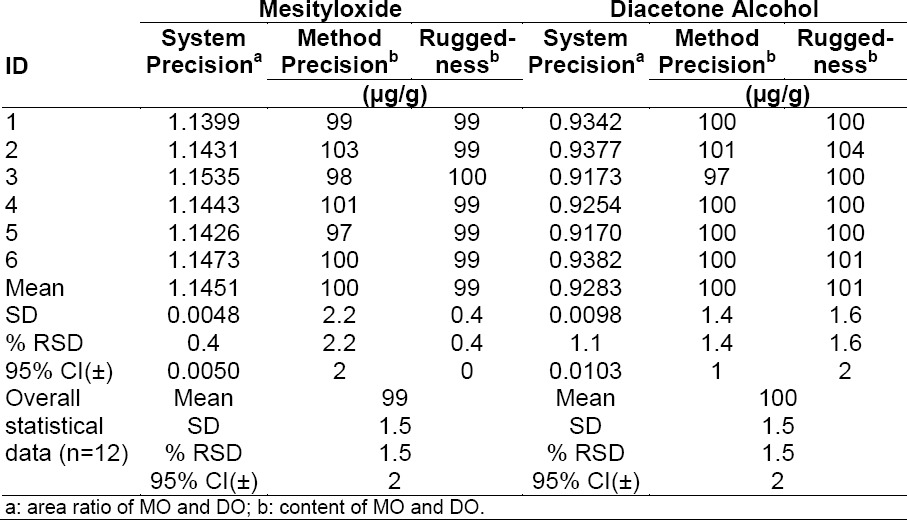
Statistical data of precision experiments

#### Robustness

This study was performed by making deliberate variations in the method parameters. The effect of variation in carrier gas flow and the column’s initial oven temperature for the MO and DO determination was studied. All experiment system suitability results (resolution between MO and DO) are mentioned in Table 6.

**Tab. 6:**
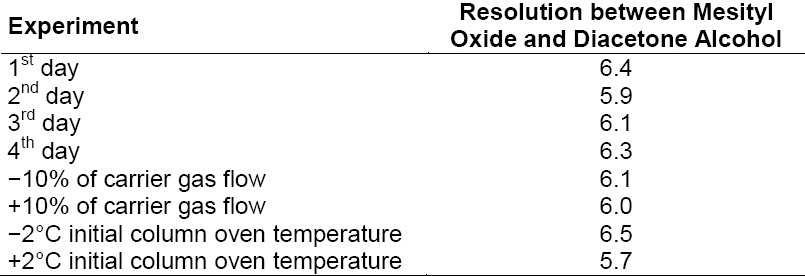
Summary of system suitability results

## Conclusion

Method validation data demonstrated that the developed GC method is sensitive. Also, the specificity of the method was established on the GC as well as accuracy for the estimation of MO and DO. Hence, the validated GC method can be employed in the routine analysis for the quantification of mesityl oxide and diacetone alcohol in atazanavir sulfate drug substance.

## References

[1] Fukushima K, Terasaka S, Haraya K, Kodera S, Seki Y, Wada A, Ito Y, Shibata N, Sugioka N, Takada K (2007). Pharmaceutical approach to HIV protease inhibitor atazanavir for bioavailability enhancement based on solid dispersion system. Biol Pharm Bull.

[2] Croom KF, Dhillon S, Keam SJ (2009). Atazanavir: a review of its use in the management of HIV-1 infection. Drugs.

[3] Swainston Harrison T, Scott LJ (2005). Atazanavir: a review of its use in the management of HIV infection. Drugs.

[4] http://packageinserts.bms.com/pi/pi_reyataz.pdf.

[5] United States Pharmacopeia–National Formulary USP37-NF32 S1, <467>Residual solvents.

[6] Inert Reassment –mesityl oxide http://www.epa.gov/opprd001/inerts/mesityl.pdf.

[7] Conant JB, Neal T, Adams R, Heckel HCN (1921). Mesityl oxide. Org Synth.

[8] Fore SP, Dupuy HP, Rayner ET (1975). Determination of mesityl oxide and diacetone alcohol in oilseed meals and Flours. J Am Oil Chem Soc.

[9] Fleet B, Jee RD, Little CJ (1973). A polarographic study of mesityl oxide and its determination in diacetone alcohol by fast-scan, phase-sensitive, A.C polatography. J Electroanal Chem Interfacial Electrochem.

[10] ICH (2005). Harmonised tripartite guideline, Validation of analytical procedures: Text and Methodology Q2(R1).

